# Does Parental Mediation of Technology Use Moderate the Associations between Cyber Aggression Involvement and Substance Use? A Three-Year Longitudinal Study

**DOI:** 10.3390/ijerph16132425

**Published:** 2019-07-08

**Authors:** Michelle F. Wright, Sebastian Wachs

**Affiliations:** 1 Department of Psychology, Pennsylvania State University, State College, PA 16802, USA; 2 Faculty of Social Studies, Masaryk University, 60200 Brno, Czech Republic; 3 Department of Educational Studies, University of Potsdam, 14476 Potsdam, Germany

**Keywords:** cyber aggression, cyber victimization, parental mediation, substance use

## Abstract

The goal of this three-year longitudinal study was to examine the buffering effect of parental mediation of adolescents’ technology use (i.e., restrictive, co-viewing, and instructive) on the relationships among cyber aggression involvement and substance use (i.e., alcohol use, marijuana use, cigarette smoking, and non-marijuana illicit drug use). Overall, 867 (*M*_age_ = 13.67, age range from 13–15 years, 51% female, 49% White) 8th grade adolescents from the Midwestern United States participated in this study during the 6th grade (Wave 1), 7th grade (Wave 2), and 8th grade (Wave 3). Results revealed that higher levels of Wave 2 instructive mediation weakened the association between Wave 1 cyber victimization and Wave 3 alcohol use and Wave 3 non-marijuana illicit drug use. The relationship was stronger between Wave 1 cyber victimization and Wave 3 alcohol use and Wave 3 non-marijuana illicit drug use when adolescents reported lower levels of Wave 2 instructive mediation. At lower levels of Wave 2 instructive mediation, the association between Wave 1 cyber aggression perpetration and Wave 3 non-marijuana illicit drug use was stronger. Implications of these findings are discussed in the context of parents recognizing their role in helping to mitigate the negative consequences associated with adolescents’ cyber aggression involvement.

## 1. Introduction

### 1.1. Cyber Aggression and Cyber Victimization

Defined as perpetrating or being targeted by purposefully hostile, embarrassing, intimidating, harassing, outing, and social exclusionary behaviors, cyber aggression is typically perpetrated with malicious intent through information and communication technologies, including email, online gaming, gaming consoles, mobile devices, instant messenger, and social networking websites [1,2,3,4,5,6,7]. Cyber aggressors have the opportunity to victimize others through information and communication technologies at almost any time and often without concern for the consequences of their actions [8,9]. The anonymity of the online environment can trigger adolescents’ inability to constrain or restrain themselves, making them vulnerable to cyber aggression perpetration or cyber victimization [10,11]. Researchers, teachers, parents, and the general public are concerned with cyber aggression involvement because of the associated negative adjustment difficulties, such as depression, anxiety, loneliness, substance use, and poor academic performance (e.g., [12,13,14,15,16]).

### 1.2. Substance Use

Several studies found that substance use is a negative outcome associated with adolescents’ involvement in cyber aggression (e.g., [14,17,18,19,20]). Research focused on substance use and cyber aggression involvement are warranted because adolescents who are involved in cyber aggression might be vulnerable to utilizing negative coping strategies, like relying on alcohol and drugs, to diminish their negative feelings resulting from being victimized or experiencing socioemotional difficulties, like depression and anxiety [21]. Many of these earlier studies on cyber aggression involvement and substance use combine different substances (e.g., alcohol, marijuana, and cigarette smoking) into one category. In one study by Hinduja and Patchin [14], they found that cyber aggression perpetration and cyber victimization were associated positively with substance use, which they measured by combining alcohol use and marijuana use into one substance use variable. Gamez-Guadix and colleagues [17] examined the longitudinal associations between cyber aggression involvement and substance use. Although they found that these variables were concurrently related, cyber aggression involvement and substance use were unrelated when investigated over six months. Like Hinduja and Patchin [14], Gamez-Guadix et al. [17] combined different types of substances, specifically tobacco, alcohol, marijuana, cocaine, speed, LSD, ecstasy, and hashish, into one variable. An explanation for these findings might be that adolescents’ cyber aggression perpetration and cyber victimization differentially impact their substance use. To address this proposal, Wright [20] examined the association of cyber victimization to adolescents’ alcohol use, marijuana use, cigarette smoking, and non-marijuana illicit drug use, assessed over three years. Findings revealed that cyber victimization was related positively to alcohol use and non-marijuana drug use. Despite adding valuable knowledge to the literature, Wright [20] did not examine cyber aggression perpetration, which can also increase adolescents’ risk of using different types of substances. Since cyber aggression involvement can increase adolescents’ risk of substance use, it is important that research also focus on the variables that might buffer against the harmful impacts of cyber aggression involvement. One variable proposed to buffer against the harmful effects of cyber aggression involvement is parental mediation of adolescents’ technology use.

### 1.3. Parental Mediation of Technology

Parental mediation is defined as parents’ use of strategies to manage their children’s relationship with digital media and technologies [22]. Research focused on parents’ mediation of technology indicates that such mediation has been generally considered as a useful strategy to mitigate online risks. Some parents set time limits on their children’s digital media use and the type of content that their children are allowed to view on the Internet [23]. However, many of these rules do not involve cyber aggression. Research has revealed that parental mediation of technology use reduces adolescents’ risk of experiencing cyberbullying, particularly when parents monitored their children’s internet use and set rules regarding the types of websites their children were allowed to visit [24]. Creating rules about the amount of time that adolescents can spend online and employing monitoring software reduced the amount of personal information adolescents shared online, which subsequently diminished their cyber aggression involvement [25]. Much of the studies on parental mediation of technology use involve reducing adolescents’ vulnerability to cyber aggression [22,23,24,26]. Little attention has been given to the buffering effect of parental mediation on the negative adjustment difficulties associated with cyber aggression involvement. In one study on this topic, Wright [27] found that there was a weaker association between cyber victimization and depression and anxiety when parents employed parental mediation of technology. In her study, Wright focused on only one type of parental mediation, despite there being different types of parental mediation.

Parental mediation strategies can include restrictive, co-viewing, and instructive strategies [28]. Restrictive mediation is defined as parents preventing their children from accessing specific online content. Co-viewing mediation is defined as parents and their children accessing online content together, although parents might not necessarily discuss content with their children. Instructive mediation is defined as parents engaging in active discussion with their children regarding online content. Some research found that restrictive mediation is associated positively whereas co-viewing mediation and instructive mediation are associated negatively with cyber victimization [20]. Furthermore, co-viewing mediation and instructive mediation weakened the negative association between adolescents’ cyber victimization and depression and anxiety. It is, however, unclear whether different technology mediation strategies could impact the relationship between cyber aggression involvement and substance use. Furthermore, much of the previous research is cross-sectional, with few longitudinal studies linking cyber aggression involvement, parental mediation, and substance use.

Lower levels of parental monitoring and mediation increase adolescents’ involvement in various antisocial and delinquent behaviors [29,30,31,32]. Given this literature, it is important to understand how parents might influence the linkages between cyber aggression involvement and substance use, especially considering that cyber aggression involvement and substance use are associated with each other [20]. Parental mediation might have a profound impact on these relationships, warranting the need for the present study.

### 1.4. The Present Study

In sum, there are gaps in the literature regarding the role of parental mediation of technology use as a buffer against the negative outcomes associated with adolescents’ cyber aggression and cyber victimization. Similarly, little is known about how different parental mediation strategies impact these associations and whether such impacts might differ when considering various types of substance use variables. The purpose of the present study was to examine the three-year longitudinal buffering effect of Wave 2 (7th grade) parental mediation strategies (i.e., restrictive, co-viewing, and instructive) in the relationship between adolescents’ Wave 1 (6th grade) cyber aggression involvement (i.e., perpetration, and victimization) and Wave 3 (8th grade) substance use (i.e., alcohol, cigarette smoking, marijuana, and non-marijuana illicit drugs). Face-to-face aggression and victimization were controlled in order to account for the positive associations between these variables and cyber aggression involvement [13,21,23,33]. To address the study’s purpose, the following research question was investigated: What, if any, buffering effect do parental mediation strategies have on the longitudinal associations between cyber aggression involvement and substance use, while controlling for face-to-face aggression and victimization?

## 2. Materials and Methods

### 2.1. Participants

There were 867 8th graders (51% female; age range = 13–15 years old; *M*_age_ = 13.67) who participated in this research. Their schools were located in the suburbs of a large Midwestern city. Adolescents self-identified as White (49%), Latino/Latina (30%), Black/African American (10%), Asian (7%), and biracial (4%). The schools were located within predominantly middle-class neighborhoods. Around 10% to 32% of students at the middle schools qualify or receive free or reduced-price lunch.

### 2.2. Procedures

The study was approved through the university’s institutional review board (IRB). A list of more than 200 middle schools was generated for the purpose of recruitment. Of these 200 middle schools, 15 schools were randomly selected. Recruitment messages were sent via email to the school principals. The recruitment message described the nature of the study, what adolescents would be expected to do, and how long adolescents would participate in the study. Out of the 15 schools, six middle schools were able to commit to the three-year data collection timeline. From these six schools, three required district level approval through their school-district IRB, which was obtained. The other three schools did not require district-level approval, and instead principals provided their approval and site permission. Announcements were made to all 6th grade classrooms, which explained the purpose of the study, why adolescents were being invited to participate, what they would do during the study, and how long they would participate in the study. Letters and parental permission slips were distributed to adolescents. They were tasked to return the parental permission slips to their school within one week. There were 1197 parental permission slips distributed. Of the parental permission slips, 1003 were returned, with 26 not consenting. This resulted in a sample size of 977 at Wave 1 in the 6th grade. Prior to completing the surveys, adolescents were asked to provide their assent to participate in the study. Assent was given by all adolescents at Wave 1.

One year after Wave 1, Wave 2 (in 7th grade) was collected and then Wave 3 (in the 8th grade) was collected two years later. For Wave 2 and Wave 3, a reminder letter was sent home to adolescents’ parents or guardians to remind them about the study. Parents/guardians were asked to write their child’s name on the letter, if they no longer wanted their child to be part of the study. At Wave 2, five parents/guardians returned the letter to their child’s school indicating that they no longer wished for their child to be enrolled in the study. There were 48 adolescents who were dropped from the study during Wave 2, either because their parents no longer wished for them to participate, they were absent on the day of data collection and the make up day, or they had moved away. During data collection at Wave 2, five adolescents did not want to participate, making a final sample of 924 at Wave 2. For Wave 3, there were no parent/guardian letters returned. There were 57 participants who were no longer available because they were absent on the day of data collection and the makeup day or they had moved away. Thus, the final sample at Wave 3 was 867.

During data collection, research personnel were present to answer any questions that adolescents had. Before completing the questionnaires, adolescents answered demographic questions concerning their age, gender, and ethnicity. Adolescents completed the following questionnaires: Self-reported cyber victimization (Wave 1 only), self-reported cyber aggression perpetration (Wave 1 only), self-reported face-to-face victimization (Wave 1 only), self-reported face-to-face aggression perpetration (Wave 1 only), perceived parental mediation of technology use (Wave 1 and Wave 2), and substance use (Wave 1, Wave 2, and Wave 3).

### 2.3. Measures

#### 2.3.1. Self-Reported Face-to-Face and Cyber Aggression Perpetration and Victimization

Adolescents were asked to report how often they experienced cyber victimization or perpetrated cyber aggression online or through text messages within the past 60 days [16]. They were also asked to indicate how often they were victimized or perpetrated face-to-face aggression within the past 60 days [34]. There were four subscales for this questionnaire: Cyber victimization, cyber aggression perpetration, face-to-face victimization, and face-to-face aggression perpetration. There were 10 items for each subscale, resulting in a total of 40 items included for this questionnaire, which were rated on a scale of 1 (never) to 9 (daily). Examples included having mean or hurtful messages sent to them online or through text messages/sending mean or hurtful messages to others online or through text messages and having someone threaten to harm them/threatening to harm someone else. All items were averaged to form four final scores on cyber victimization, cyber aggression perpetration, face-to-face victimization, and face-to-face aggression perpetration. Cronbach’s alpha was 0.88 for cyber victimization, 0.86 for cyber aggression perpetration, 0.89 for face-to-face victimization, and 0.86 for face-to-face aggression perpetration. This questionnaire was administered at Time 1 only.

#### 2.3.2. Perceived Parental Mediation of Technology Use

For this questionnaire, adolescents were asked to indicate how much they agree or disagree that their parents are involved in their technology use, using a scale of 1 (completely disagree) to 5 (completely agree) [28]. The questionnaires included nine items, with three subscales: Restrictive mediation (four items; e.g., “My parents impose a time limit on the amount of the time that I surf the Internet”), co-viewing mediation (three items; e.g., “My parents surf the internet with me”), and instructive mediation (two items; e.g., “My parents show me how to use the Internet and warn me about its risks”). Cronbach’s alphas were 0.90 for restrictive mediation, 0.86 for co-viewing mediation, and 0.80 for instructive mediation at Wave 1. At Wave 2, Cronbach’s alphas were 0.89 for restrictive mediation, 0.86 for co-viewing mediation, and 0.81 for instructive mediation. This questionnaire was completed at Wave 1 and Wave 2. Parents’ perceptions of their own mediation of technology use were not examined because parents often overestimate how often they monitor and set rules for their children’s technology use [24,35].

#### 2.3.3. Substance Use

This questionnaire asked adolescents to report how often within the past 12 months they used different substances. The substances were organized into four categories: Alcohol use (one item), cigarette smoking (one item), marijuana use (one item), and non-marijuana illicit drug use (five items; i.e., cocaine, hallucinogens, heroin, inhalants, and prescription drugs). The items were rated on a scale of 1 (never in the past 12 months) to 5 (every day or almost every day). The five items that made up the non-marijuana illicit drug use variable were combined to form a final score for non-marijuana illicit drug use, resulting in a Cronbach’s alpha of 0.79 at Wave 1, 0.80 at Wave 2, and 0.79 at Wave 3.

### 2.4. Analytic Plan

The software program *Mplus 7.3* [36] was used to conduct the structural regression model, before testing associations among the variables. The model fit was adequate, χ2 = 791.93, df = 888, p = 0.99, comparative fit index (CFI) = 0.98, Tucker Lewis index (TLI) = 0.97, root mean square error of approximation (RMSEA) = 0.03, standardized root mean square residual (SRMR) = 0.04. Standardized factor loadings had adequate magnitudes and significant factor loadings, *ps* < 0.001. Items for each questionnaire served as indicators for the latent variables in the structural regression model. Paths were included from Wave 1 cyber victimization and Wave 1 cyber aggression perpetration to the three Wave 2 parental mediation strategies (i.e., restrictive, co-viewing, and instructive), and from these three Wave 2 parental mediation strategies to Wave 3 substance use (i.e., alcohol use, cigarette smoking, marijuana use, and non-marijuana illicit drug use). There were also paths specified from three Wave 2 parental mediation strategies to Wave 3 substance use. The three Wave 1 parental mediation strategies were also allowed to predict their three respective Wave 2 parental mediation strategies. Similarly, Wave 1 and Wave 2 substance use variables were specified to predict Wave 3 substance use variables. To control for Wave 1 face-to-face victimization and Wave 1 face-to-face aggression perpetration, these variables were allowed to predict Wave 1 cyber victimization and Wave 1 cyber aggression perpetration. Six interactions were also included in the model between Wave 1 cyber victimization and Wave 2 restrictive mediation, Wave 1 cyber aggression perpetration and Wave 2 restrictive mediation, Wave 1 cyber victimization and Wave 2 co-viewing mediation, Wave 1 cyber aggression perpetration and Wave 2 co-viewing mediation, Wave 1 cyber victimization and Wave 2 instructive mediation, and Wave 1 cyber aggression perpetration and Wave 2 instructive mediation. The model without gender fit the data well, *χ*2 (623) = 701.58, *p* = 0.02, *CFI* = 0.99, *TLI* = 0.98, *RMSEA* = 0.04, and *SRMR* = 0.05.

## 3. Results

Means and standard deviations were computed for all variables included in the study (see Table 1).

Correlations were performed among all variables (see Table 2).

Wave 1 face-to-face victimization, Wave 1 face-to-face aggression perpetration, Wave 1 cyber victimization, and Wave 1 cyber aggression perpetration were correlated with Wave 1 alcohol use, Wave 1 cigarette smoking, Wave 1 non-marijuana illicit drug use, Wave 2 alcohol use, Wave 2 cigarette smoking, Wave 2 non-marijuana illicit drug use, Wave 3 alcohol use, Wave 3 cigarette smoking, and Wave 3 non-marijuana illicit drug use. Wave 1 restrictive, co-viewing, and instructive mediation were correlated with Wave 1 through Wave 3 alcohol use and Wave 1 through Wave 3 non-marijuana illicit drug use. Wave 2 restrictive mediation was correlated with Wave 1 through Wave 3 non-marijuana illicit drug use and Wave 3 alcohol use. Wave 2 co-viewing mediation was correlated with Wave 2 and Wave 3 alcohol use and Wave 1 through Wave 3 non-marijuana illicit drug use. Wave 2 instructive mediation correlated with Wave 1 through Wave 3 alcohol use and Wave 1 through Wave 3 non-marijuana illicit drug use.

### 3.1. Main Effects of Cyber Victimization, Cyber Aggression Perpetration, and Parental Mediation

All Wave 1 and Wave 2 substance use variables, including alcohol use, cigarette smoking, marijuana use, and non-marijuana illicit drug use were related to each of the Wave 3 substance use variables (see Table 3).

Wave 1 cyber victimization and Wave 1 cyber aggression perpetration were also associated positively with Wave 3 alcohol use, cigarette smoking, marijuana use, and non-marijuana illicit drug use. Wave 2 restrictive, co-viewing, and instructive mediations were related negatively to Wave 3 alcohol use. Wave 2 instructive mediation was associated negatively with Wave 3 marijuana use. Similarly, Wave 2 instructive mediation was related negatively to Wave 3 non-marijuana illicit drug use.

### 3.2. Moderation of Parental Mediation

Significant interactions were found between Wave 1 cyber victimization and Wave 2 instructive mediation when predicting Wave 3 alcohol use and Wave 3 non-marijuana illicit drug use, and between Wave 1 cyber aggression perpetration and Wave 2 instructive mediation when predicting Wave 3 non-marijuana illicit drug use. The Interaction Program to probe the interaction further by graphing the representation of the interaction and calculating the significance of the simple slopes. Wave 1 cyber victimization was associated with greater Wave 3 alcohol use and Wave 3 non-marijuana illicit drug use when adolescents reported lower levels of Wave 2 instructive mediation (*B* = 0.02, *SE* = 0.02, *p* = n.s. at the mean, *B* = 0.05, *SE* = 0.03, *p* < 0.01 at −1 *SD* for Wave 3 alcohol use; *B* = 0.01, *SE* = 0.01, *p* = n.s. at the mean, *B* = 0.06, *SE* = 0.02, *p* < 0.01 at −1 *SD* for Wave 3 non-marijuana illicit drug use). Wave 1 cyber victimization and Wave 3 alcohol use and Wave 3 non-marijuana illicit drug use were less associated at higher levels of Wave 2 instructive mediation (*B* = −0.06, *SE* = 0.04, *p* < 0.01 at + 1 *SD* for Wave 3 alcohol use; *B* = −0.08, *SE* = 0.04, *p* < 0.001 at + 1 *SD* for Wave 3 non-marijuana illicit drug use). At lower levels of Wave 2 instructive mediation, the association between Wave 1 cyber aggression perpetration and Wave 3 non-marijuana illicit drug use was more positive (*B* = −0.01, *SE* = 0.01, *p* = *n.s*. at + 1 *SD*, *B* = 0.01, *SE* = 0.02, *p* = *n.s.* at the mean, *B* = 0.04, *SE* = 0.02, *p* < 0.05 at −1 *SD*).

## 4. Discussion

The goal of this three-year longitudinal study was to investigate whether Wave 2 parental mediation of adolescents’ technology use could buffer or weaken the relationship between cyber aggression involvement in 6th grade (Wave 1) and substance use (i.e., alcohol, marijuana, cigarette smoking, and non-marijuana illicit drugs) in 8th grade (Wave 3). Wave 1 parental mediation strategies and Wave 1 and Wave 2 substance use were controlled for in all analyses. This study contributes to the literature by finding that substance use was related to cyber aggression involvement. It also adds to the literature by focusing on factors that might mitigate the negative adjustment difficulties, specifically substance use, associated with cyber aggression perpetration and cyber victimization. Victims of cyber aggression might use substances to self-medicate their feelings of depression and anxiety after being victimized, using such substances as a mechanism to cope with what has happened to them [37,38,39]. Cyber aggression perpetrators may use substances due to impulsivity and to deal with stress and mental health issues that might have led them to harm others through information and communication technologies [40,41]. Furthermore, this study addresses limitations of previous research by utilizing a longitudinal design, controlling for both face-to-face aggression perpetration and victimization, and examining different types of substances.

Parental mediation of adolescents’ technology use the might function as a form of social support, which enables adolescents to discuss their exposure to negative online situations with their parents [42]. Furthermore, parental mediation involves parents discussing strategies with their children on ways to mitigate or reduce exposure to online risks [27]. Such discussions might make adolescents feel like what happens to them matters, and as a result they might be more willing to seek out support and guidance from their parents regarding online experiences. Seeking out such support might increase when adolescents experience technical problems or uncomfortable situations while using information and communication technologies [43,44]. The appeal of parental mediation of adolescents’ technology use as a form of social support is that social support buffers against the negative adjustment difficulties associated with both online and offline aggression [45,46]. In essence, parental mediation of technology use opens the lines of continuous communication between parents and adolescents concerning online experiences.

This study’s results are best understood through the investigation of the moderating effect of parental mediation of technology use on the relationships between cyber aggression involvement and substance use. Findings from this present study suggested that lower levels of instructive mediation in 7th grade (Wave 2) strengthened the association between cyber victimization in 6th grade and alcohol use and non-marijuana illicit drug in 8th grade, while such relationships were less positive at higher levels of instructive mediation in 7th grade. In addition, increases in instructive mediation during 7th grade increased the positive relationship between cyber aggression perpetration in 6th grade and non-marijuana illicit drug use in 8th grade. Due to the lack of research conducted on the buffering effect of parental mediation, it is difficult to reconcile these findings with the literature. Instructive mediation involves parents who openly communicate with their children about online content, which includes opportunities to discuss negative online experiences, like cyber aggression involvement, and strategies for how their children can reduce exposure to these experiences [24,28,43,44]. Adolescents whose parents utilize instructive mediation might be more adept at avoiding situations that could promote cyber aggression involvement and they might also have the skills to deal more effectively with these situations, minimizing their exposure to these experiences in the future. Research supports instructive mediation as a strategy that can help to reduce adolescents’ risk of cyberbullying and as a buffer against the depression and anxiety associated with cyberbullying [24,25,27].

Results from the present study indicated that co-viewing mediation in 7th grade did not moderate the relationships between cyber aggression involvement in 6th grade and substance use variables during 8th grade. Parents who implement co-viewing mediation do not necessarily discuss online content, even while they jointly view content with their children [28]. Such parents might not provide their children with enough knowledge regarding ways to deal with negative online situations. It is unclear how much communication occurs among parents who utilize co-viewing mediation strategies and their children. Viewing content with one’s children might not be enough to mitigate the negative consequences associated with cyber aggression involvement, though it might offer some form of social support.

Restrictive mediation in the 7th grade did not moderate the associations between cyber aggression involvement in 6th grade and substance use during 8th grade. In the literature, Mesch [24] found that restrictive mediation often involves parents implementing strict rules regarding information and communication technology use. These parents usually do not discuss ways to deal with adolescents’ exposure to unwanted and unpleasant experiences through technology. Restrictive mediation might potentially be a form of the overprotective parenting style in which parents do not allow their children to develop problem-solving abilities and social skills [47,48]. It is unlikely that parents can protect adolescents from being exposed to risky situations online by utilizing restrictive mediation because they might not allow their children to develop strategies for dealing with these situations, increasing adolescents’ risk of experiencing cyber aggression.

### Limitations and Future Research Directions

Although the present study contributes valuable insight into the role of parental mediation in the associations between cyber aggression involvement and substance use, there are some limitations that should be addressed in future research. It is difficult to determine the temporal ordering of the variables examined in this study. Therefore, follow-up research should also assess parental mediation and cyber aggression involvement at multiple waves as well. This research should also examine changes in parental mediation strategies over time by examining these strategies from childhood into adolescence. In addition, the present study used self-reports, which are susceptible to biases. Follow-up research should include multiple informants, such as parents’ reports of their own perceptions of their technology mediation. It is further important to investigate parents’ reports of their own perceived media competence as such perceptions could impact their children’s online behavior. Finally, future research should investigate whether the effects of parental mediation vary in different socioeconomic groups, and if yes to which extent.

## 5. Conclusions

This study was one of the first to focus on examining the buffering effect of parental mediation strategies on the three-year longitudinal associations among cyber aggression involvement and substance use. Results from this study suggested that higher levels of instructive mediation in 7th grade weakened the positive association between cyber victimization in 6th grade and alcohol use and non-marijuana drug use during 8th grade. Lower levels of instructive mediation in 7th grade increased the positive association between cyber victimization in 6th grade and alcohol use in 8th grade, cyber victimization in 6th grade and alcohol use in 8th grade, and cyber aggression perpetration in 6th grade and non-marijuana drug use in 8th grade less positive. These findings suggest that it is important to raise awareness of cyber aggression involvement and the possibility that some parental mediation strategies reduce adolescents’ use of substances. Parents should be encouraged to understand that they have a supportive role in adolescents’ technology use, and how they might help to diminish the negative consequences associated with cyber aggression involvement. Therefore, parents must be involved in their children’s technology use to help protect children from the substance use that might result as a consequence of cyber aggression perpetration and victimization.

## Figures and Tables

**Table 1:** Descriptive statistics for face-to-face victimization, face-to-face aggression, cyber victimization, cyber aggression, parental mediation, and substance use.

	M	SD
Wave 1 Face-to-face Victimization	6.13	1.01
Wave 1 Face-to-face Aggression	5.43	0.96
Wave 1 Cyber Victimization	5.31	0.91
Wave 1 Cyber Aggression	4.08	1.00
Wave 1 Restrictive Mediation	2.97	0.86
Wave 1 Co-viewing Mediation	2.94	0.76
Wave 1 Instructive Mediation	3.08	0.99
Wave 2 Restrictive Mediation	2.89	0.88
Wave 2 Co-viewing Mediation	2.91	0.80
Wave 2 Instructive Mediation	3.06	0.95
Wave 1 Alcohol Use	2.09	0.45
Wave 1 Cigarette Smoking	1.89	0.46
Wave 1 Marijuana Use	2.08	0.53
Wave 1 Non-marijuana illicit drug use	1.45	0.43
Wave 2 Alcohol Use	2.16	0.50
Wave 2 Cigarette Smoking	1.91	0.51
Wave 2 Marijuana Use	2.10	0.50
Wave 2 Non-marijuana illicit drug use	1.46	0.42
Wave 3 Alcohol Use	2.18	0.51
Wave 3 Cigarette Smoking	1.91	0.60
Wave 3 Marijuana Use	2.15	0.49
Wave 3 Non-marijuana illicit drug use	1.49	0.52

**Table 2:** Correlations among face-to-face victimization, face-to-face aggression, cyber victimization, cyber aggression, parental mediation, and substance use.

	W1 Alcohol Use	W1 Cigarette Smoking	W1 Marijuana Use	W1 NMID	W2 Alcohol Use	W2 Cigarette Smoking	W2 Marijuana Use	W2 NMID	W3 Alcohol Use	W3 Cigarette Smoking	W3 Marijuana Use	W3 NMID
W1 F−Vic	0.27 ***	0.16 *	0.07	0.30 ***	0.24 **	0.16 *	0.06	0.27 ***	0.26 **	0.18 *	0.08	0.28 ***
W1 F−Agg	0.28 ***	0.16 *	0.06	0.31 ***	0.19 *	0.19 *	0.06	0.27 ***	0.23 *	0.20 *	0.07	0.31 ***
W1 C−Vic	0.26 ***	0.21 *	0.08	0.30 ***	0.23 *	0.20 *	0.07	0.30 ***	0.25 **	0.21 *	0.07	0.31 ***
W1 C−Agg	0.26 ***	0.20 *	0.08	0.33 ***	0.26**	0.18 *	0.06	0.32 ***	0.26 **	0.19 *	0.05	0.32 ***
W1 R−Med	−0.20 *	−0.10	−0.03	−0.18 *	−0.19 *	−0.11	−0.03	−0.17 *	−0.22 *	−0.10	−0.01	−0.19 *
W1 C−Med	−0.17 *	−0.09	−0.02	−0.18 *	−0.20 *	−0.10	−0.01	−0.17 *	−0.20 *	−0.09	−0.01	−0.20 *
W1 I−Med	−0.26 **	−0.10	−0.05	−0.23 *	−0.23 *	−0.10	−0.04	−0.25 *	−0.22 *	−0.09	−0.02	−0.25 *
W2 R−Med	−0.15	−0.05	−0.03	−0.17 *	−0.14	−0.05	−0.04	−0.19 *	−0.17 *	−0.06	−0.05	−0.21 *
W2 C−Med	−0.16	−0.07	−0.04	−0.18 *	−0.18 *	−0.09	−0.02	−0.20 *	−0.19 *	−0.05	−0.05	−0.22 *
W2 I−Med	−0.23 *	−0.07	−0.03	−0.26 **	−0.23 *	−0.08	−0.03	−0.23 *	−0.21 *	−0.06	−0.03	−0.26 **

Note. W1 = Wave 1, W2 = Wave 2, W3 = Wave 3, NMID = non-marijuana illicit drug use, F-Vic = face-to-face victimization, F-Agg = face-to-face aggression, C-Vic = cyber victimization, C-Agg = cyber aggression, R-Med = restrictive mediation, C-Med = co-viewing mediation, I-Med = instructive mediation. * *p* < 0.05. ** *p* < 0.01. *** *p* < 0.001.

**Table 3:** Structural regression model results: Standardized regression coefficients and significance value.

	W3 Alcohol Use		W3 Cigarette Smoking		W3 Marijuana Use		W3 NMID	
	β	*p*	β	*p*	β	*p*	β	*p*
Substance Use								
W1 Alcohol Use	***0.25***	***0.001***	***0.16***	***0.011***	***0.21***	***0.001***	***0.18***	***0.007***
W1 Cigarette Smoking	***0.18***	***0.004***	***0.22***	***0.001***	***0.16***	***0.010***	***0.24***	***0.001***
W1 Marijuana Use	***0.19***	***0.006***	***0.11***	***0.029***	***0.23***	***0.001***	***0.23***	***0.001***
W1 NMID	***0.20***	***0.001***	***0.19***	***0.009***	***0.16***	***0.009***	***0.28***	***0.001***
W2 Alcohol Use	***0.23***	***0.001***	***0.11***	***0.033***	***0.14***	***0.022***	***0.15***	***0.017***
W2 Cigarette Smoking	***0.13***	***0.016***	***0.19***	***0.002***	***0.09***	***0.048***	***0.21***	***0.001***
W2 Marijuana Use	***0.18***	***0.003***	***0.09***	***0.041***	***0.19***	***0.004***	***0.19***	***0.001***
W2 NMID	***0.17***	***0.004***	***0.17***	***0.019***	***0.11***	***0.029***	***0.25***	***0.001***
Victimization & Aggression								
W1 Cyber Victimization	***0.30***	***0.001***	***0.19***	***0.001***	***0.13***	***0.023***	***0.31***	***0.001***
W1 Cyber Aggression	***0.27***	***0.001***	***0.17***	***0.001***	***0.08***	***0.041***	***0.26***	***0.001***
Parental Mediation								
W2 Restrictive Mediation	***−0.10***	***0.020***	−0.13	0.183	−0.10	0.109	***−0.13***	***0.019***
W2 Co−Viewing Mediation	***−0.13***	***0.007***	−0.04	0.769	−0.11	0.099	***−0.16***	***0.008***
W2 Instructive Mediation	***−0.25***	***0.001***	−0.03	0.746	***−0.17***	***0.001***	***−0.21***	***0.001***
Interactions								
W1 Cyber Victimization × W2 Restrictive Mediation	−0.01	0.898	−0.02	0.767	−0.01	0.812	−0.03	0.716
W1 Cyber Victimization × W2 Co−Viewing Mediation	−0.09	0.115	−0.05	0.541	−0.07	0.513	−0.02	0.745
W1 Cyber Victimization × W2 Instructive Mediation	***−0.19***	***0.039***	−0.01	0.989	−0.01	0.976	***−0.16***	***0.021***
W1 Cyber Aggression × W2 Restrictive Mediation	−0.01	0.929	−0.01	0.963	−0.02	0.943	−0.01	0.991
W1 Cyber Aggression × W2 Co−Viewing Mediation	−0.08	0.202	0.01	0.889	−0.01	0.913	−0.02	0.898
W1 Cyber Aggression × W2 Instructive Mediation	−0.06	0.212	−0.05	0.293	−0.03	0.398	***−0.12***	***0.041***

Note. W1 = Wave 1, W2 = Wave 2, W3 = Wave 3, NMID = non-marijuana illicit drug use. Bold and italicized and bold numbers indicate statistically significant findings. Not shown in the table are the relationships between W1 face-to-face victimization and W1 cyber victimization (β = 0.25, *p* = 0.012), W1 face-to-face aggression and W1 cyber aggression (β = 0.23, *p* = 0.027), W1 restrictive mediation and W2 restrictive mediation (β = 0.19, *p* = 0.039), W1 co-viewing mediation and W2 co-viewing mediation (β = 0.20, *p* = 0.026), and W1 instructive mediation and W2 instructive mediation (β = 0.23, *p* = 0.025). * *p* < 0.05. ** *p* < 0.01. *** *p* < 0.001.
